# Road Pavement Thickness and Construction Depth Optimization Using Treated and Untreated Artificially-Synthesized Expansive Road Subgrade Materials with Varying Plasticity Index

**DOI:** 10.3390/ma15082773

**Published:** 2022-04-09

**Authors:** Samuel Y. O. Amakye, Samuel J. Abbey, Colin A. Booth, Jonathan Oti

**Affiliations:** 1 Faculty of Environment and Technology, University of the West of England, Bristol BS16 1QY, UK; samuel.abbey@uwe.ac.uk (S.J.A.); colin.booth@uwe.ac.uk (C.A.B.); 2 School of Engineering, Faculty of Computing, Engineering and Science, University of South Wales, Pontypridd CF37 1DL, UK; jonathan.oti@southwales.ac.uk

**Keywords:** expansive subgrade material, artificially-synthesized subgrade, California bearing ratio, road pavement thickness optimization, compaction test, swell test

## Abstract

Road pavement thickness and their depth of construction take a chunk of the overall cost of road construction. This has called for a need for reduced road pavement thickness by improving the engineering properties of subgrade such as the California bearing ratio (CBR). The CBR of road subgrade has been a major determining factor for road pavement thickness, and expansive subgrades generally have a low CBR, resulting in major road defects. In this study, road pavement thickness and construction depth optimization were conducted using the CBR values achieved in this study. Additives proportions of 8% lime and 20% cement were used in expansive subgrade to improve their engineering properties, making them suitable for use in road construction. The study investigated the characteristics, mineral structure, Atterberg limit, compaction, CBR, swell and microstructural properties of expansive subgrade. The results show a reduction in road pavement thickness and a construction depth with an increase in CBR value. All CBR values for treated samples were above 2%, making them usable in road construction. A reduction in swell potential up to 0.04% was observed for treated expansive subgrade. The study concluded that pavement thickness and construction depth can be reduced by enhancing subgrade materials and using cement and lime as binders.

## 1. Introduction

Road pavements are structures that consist of superimposed layers of processed materials placed over the natural subgrade. The primary function of road pavement is to distribute traffic load to the subgrade and provide a surface of acceptable riding quality, adequate skid resistance and low noise pollution [1]. During road construction, a huge sum of the total construction cost goes into road pavement construction, especially in situations where weak or expansive subgrade is involved. The California bearing ratio (CBR) value of road pavement subgrade can influence the overall thickness and depth of the construction of road pavement, which can greatly impact the coverall construction cost [2]. Subgrade material is the natural soil underneath a road pavement structure [2]. California bearing ratio (CBR) is a penetration test to evaluate the strength of road subgrade materials to ascertain their bearing capacity for use as road subgrade materials during construction [2]. When soils exhibit evident volume changes with the potential to swell and shrink with changes in moisture content due to the presence of clay minerals, they are referred to as expansive subgrade [2]. Expansive subgrade materials do not have the capacity to support the weight of road pavement and traffic load and will normally require some form of modification or re-engineering to enhance their capacity to support the load [2]. Many road pavement defects and failures are a result of expansive subgrade, and the process of repairing or maintaining these defects comes with a huge cost and sometimes requires a total reconstruction of the road [3]. Infrastructure built on expansive soils may experience structural failure or deformation, resulting in a combined annual repair and maintenance cost of 30 billion USD to the United States and China [4]. The UK economy, over the past ten years, has suffered a cost of over 3 billion GBP, making it the most damaging geohazard in Britain [5]. According to [6], the damage caused by expansive subgrade materials in road structures runs into millions of dollars compared to the damages caused by floods.

In this study, road pavement thickness and pavement depth optimization was carried out in accordance with the CBR, which is method recommended by the California state of highways to determine how varying CBR values affect road pavement thickness using laboratory artificially-synthesized subgrade (ASS) material. The aim of this study is to determine the effect of treated and untreated expansive road subgrade materials and how they affect their CBR values and pavement thickness using chemical stabilization techniques. Chemical subgrade stabilization is an effective technique to improve expansive subgrade, and it involves adding different types of admixtures such as lime and cement, among others, as binders to stabilize soil [7,8]. Chemical soil stabilization techniques have been reportedly used in addressing the problems associated with expansive subgrades [9,10]. The addition of these chemical binders changes the gradation and physico-synthetics within and around the soil particles, promoting cation exchange, which leads to the flocculation and agglomeration of the expansive soil particles [11]. In this study, the artificially-synthesized subgrade (ASS) materials used in this study are a mixture of untreated bentonite and kaolinite clays at various percentages to form subgrade materials with the properties of an expansive subgrade similar to that of a naturally existing expansive clay subgrade material. Atterberg limits, a compaction behavior test, was conducted on untreated bentonite and kaolinite clay soil before mixing them to determine their behavior, characteristics and strength at different moisture contents. The ASS was later treated using cement and lime to improve its strength for use as subgrade materials in road construction. The California bearing ratio (CBR) test was conducted for untreated and treated ASS to determine the strength and bearing capacity for use as road subgrade materials.

**Figure 1:**
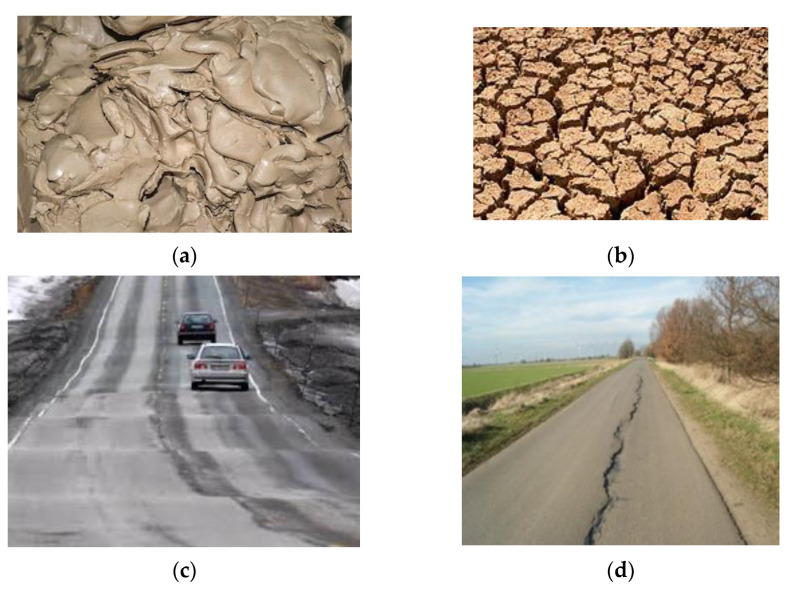
(**a**) Typical wet expansive soil [3]; (**b**) typical dry expansive soil [3]; (**c**) uplifting of flexible pavement [3]; (**d**) typical longitudinal crack on road pavement dude to expansive subgrade [3].

Cement is a finely ground powder that becomes solid when mixed with water through a process called hydration [2,12]. Over the years, Portland cement and lime have been used to improve the engineering properties of subgrade materials. Portland cement is a hydraulic binder derived through the crushing, milling and proportioning of raw material such as calcareous limestone/chalk rock and clay/shale after burning them in a large rotary kiln at a temperature of up to 1450 °C or 2600 °F. Cement solidifies when mixed with water through a process known as hydration [2]. Hydration is a chemical combination of Portland cement compounds and water to form sub-microscopic crystals. During the hydration process, a cement gel matrix is produced called calcium silicate hydrate (C-S-H), which binds subgrade particles together and is responsible for strength gain [2]. According to [13], cement is suitable for the stabilization of subgrade materials with low plasticity indexes. Cement is popularly used to improve the engineering properties of expansive subgrade materials [14]. A cement range of 4 to 15% was used to enhance the engineering properties of the subgrade materials [15]. The addition of 3% cement with 1% nano-silica and nano-alumina resulted in a 196% and 164% increase in the soaked CBR of the nontreated clay [16]. Cement, fly ash, bituminous, rice husk ash, lime, construction and demolition waste, electrical and thermal waste, geotextile fabrics and recycled waste can be used as admixtures in this process [8]. The addition of these materials as admixtures can alter the geotechnical properties of expansive soil such as the strength, bearing capacity, hydraulic conductivity, compressibility, workability, durability and swelling potentials [17]. Lime was mostly used in subgrade stabilization before the introduction of cement, and it has proven to be an effective modification agent for the stabilization of highway and airport pavement subgrade. A lime soil reaction takes place when soil mixed with lime changes the moisture and density relationship of the soil. This reaction triggers a lime hydration process and, with the help of calcium, releases cementitious products (calcium-silicate-hydrate (C-S-H) and calcium-aluminate-hydrate (C-A-H)) responsible for the strength increase in the subgrade [2,14]. The use of limestone, which is a source of lime, as road fill is very important due to the ability of limestone to improve the bearing capacity of road subgrade during the formation of C-S-H gel in the lime hydration process [18,19]. An investigation into the application of the stabilization of wastewater sludge proves that cement, lime and bitumen can be used as subgrade materials [20]. During chemical road subgrade stabilization, the shear strength of the expansive subgrade improves when stabilizers react with water within the soil, leading to an increase in stiffness of the soil [7]. A further increase in strength and durability is observed depending on the curing time and temperature [2]. In civil engineering applications, subgrade materials with a plasticity between 20% and 30%, with a liquid limit from 25% to 50%, are recommended for lime stabilization [21]. Good CBR and swelling results were achieved when 80% lime was used in the expansive subgrade stabilization for flexible pavement, and 3–8% of lime was used to improve high plasticity clays [22]. Reference [23] used 1% of lime for every 10% of clay content in the soil. Reference [24] used 6% of lime to stabilize expansive subgrade. A lime proportion of 4–6% was adopted to achieve the best performance of expansive subgrade material [25]. The treatment of expansive subgrade using lime and other additives to improve its engineering properties has been effective for road pavement construction [26]. The addition of 8% lime to an expansive black clay mixture fell beyond the satisfactory range for use as sub-base materials for light-traffic roads [27]. Black cotton soil (BCS) stabilized with 3% lime + 15% volcanic ash (VA), which meets the performance requirements of roadbed materials [28]. The inclusion of cement increased the bearing capacity of subgrade material during subgrade stabilization at proportions of 10%, 15% and 20% [29]. Subgrade materials were improved by achieving a compressive strength from 564.78 kPa to 636.19 kPa [30]. Hydraulic lime is produced by burning a form of low-grade limestone containing silica and alumina, which are above certain temperatures, combined with calcium oxide. Lime is one of the most common binders used in road subgrade stabilization [2]. According to [31], an optimum lime dosage between 6–12% by dry weight is suitable to enhance the engineering properties of road subgrade materials. Figure 1a–d shows typical wet and dry expansive soil and road pavement defects caused by expansive subgrade. Table 1 shows the advantages of treating expansive subgrade and the disadvantages of the removal and replacement of weak subgrade.

## 2. Materials and Methods

**Figure 2:**
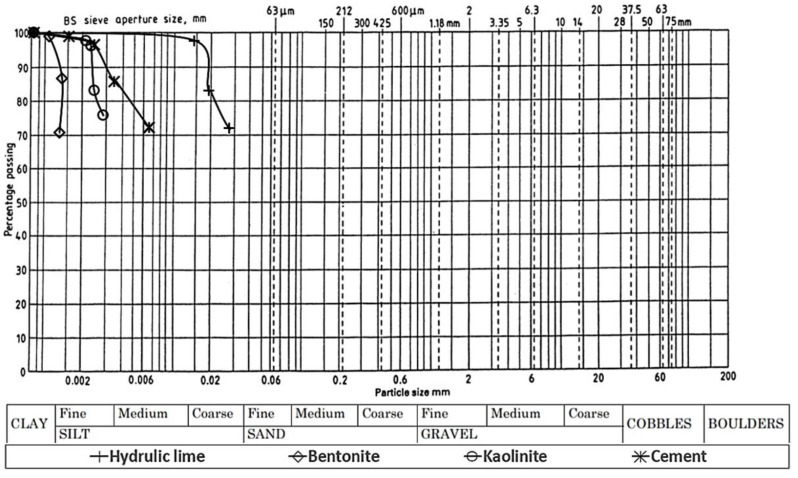
Particle size distribution of materials used in this study.

Materials used in this study consist of bentonite clay and kaolinite clay to form subgrade 1 = ASS 1 (25% bentonite, 75% kaolinite), subgrade 2 = ASS 2 (35% bentonite, 65% kaolinite) and subgrade 3 = ASS 3 (75% bentonite, 25% kaolinite), respectively. The kaolinite used was supplied by Potclays Ltd. Brickkiln Lane, Etruria, Stoke-on-Trent, England, and the bentonite used was supplied by Potclays Ltd., Brickkiln Lane, Etruria, Stoke-on-Trent, England. The cement used (CEM I) complies with BS EN 197-1:2011 [32] and was supplied by CEMEX UK Operations Ltd., CEMEX House, Evreux Way, Rugby, Warwickshire, CV21 2DT, and the lime used was (quicklime), complies with BS EN 459-1-2015 [33] and was supplied by Singleton Birch Ltd., Melton Ross Quarries, Barnetby, North Lincolnshire. Table 2 and Table 3 show the oxide and some of the chemical and mineralogical composition of bentonite and kaolinite. Table 4 shows consistency limits and physical properties of kaolinite and bentonite used in this study. Figure 2 shows the particle size distribution of bentonite, kaolinite, cement and lime used in this study.

**Figure 3:**
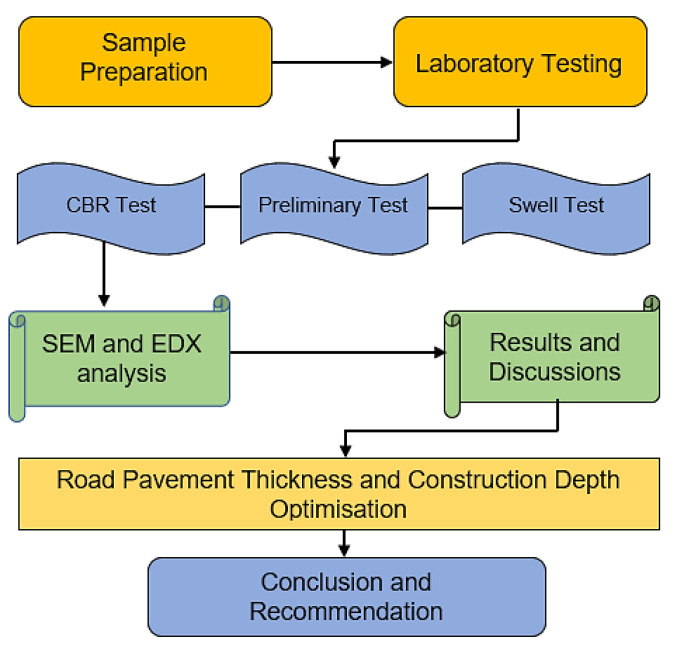
Methodological process.

This study focused on road pavement thickness optimization using treated artificially-synthesized expansive subgrade composed of a mixture of bentonite and kaolinite at various percentages, in accordance with BS 1924-1:2018 [34]. The Atterberg limits and the compaction behavior of untreated bentonite and kaolinite clay were investigated. The California bearing ratio (CBR) for treated and untreated ASS was conducted to ascertain the strength and bearing capacity of treated expansive soil for use as road subgrade materials in road construction. SEM and EDX analyses were conducted for treated subgrade materials to see how the addition of binders affected the engineering properties of the subgrade materials. Based on the CBR values achieved in the laboratory test, pavement thickness and construction depth optimization were carried out in accordance with the CBR method recommended by the California state of highways to determine the effect of varying CBR values on pavement thickness and construction depth. The methodological process used to achieve the aim of this study is illustrated in Figure 3.

Bentonite and kaolinite clays were mixed in various proportions by the weight of soil to form subgrade 1 = ASS 1(25% bentonite, 75% kaolinite), subgrade 2 = ASS 2 (35% bentonite, 65% kaolinite) and subgrade 3 = ASS 3 (75% bentonite, 25% kaolinite). Compaction and Atterberg limit tests for the various proportions of the ASS were conducted in accordance with BS EN ISO 17892-1-2014 [35], BS EN ISO 17892-12:2018+A1:2021 [36], BS 1377-4:1990 [37], AASHTO T265 [38], ASTM D2216-19 [39], ASTM D4318-17e1 [40], AASHTO T90 [41] and AASHTO T89 [42] to determine their optimum moisture content (OMC) and maximum dry density (MDD). 

### 2.1. California Bearing Ratio (CBR)

The California bearing ratio is a penetration test conducted to evaluate the strength and bearing capacity of road subgrade material. Knowing the CBR value of subgrade material prior to any road construction influences the design and construction of road pavement. According to BS EN 13286-47:2021 [43], the classification of a road in terms of pavement thickness and how much traffic a road can carry are dependent on the CBR value of the subgrade material. A typical CBR value of 2% equates to clay, while some sands may have a CBR value of 10%. A high-quality subgrade normally has a CBR value between 80–100% maximum [44]. Relevant road pavement design guidance documents such as the Design Manual for Roads and Bridges (DMRB) and the Indian Roads Congress—IRC-37-2001 [45] state that the higher the CBR value the thinner the road pavement, and the lower the CBR value the thicker the road pavement. During road pavement design, thicker pavements are recommended to compensate for the low CBR value of weak road subgrade material to enable it to carry a traffic load. According to [46], a document on flexible pavement design by the California bearing ratio method showed that a CBR value of 2% will require a road pavement thickness of 700 mm to carry heavy traffic (5443 kg), while a CBR value of 80% will require a pavement thickness of 70 mm to withstand a heavy traffic load (5443 kg). A subgrade with a CBR value < 2% is unacceptable for road construction and will require engineering or modification to make it suitable for use in road construction [46]. This means that CBR values affect the design, overall thickness and cost of road construction and has to be taken seriously during a road project.

#### CBR Sample Preparation and Testing 

**Figure 4:**
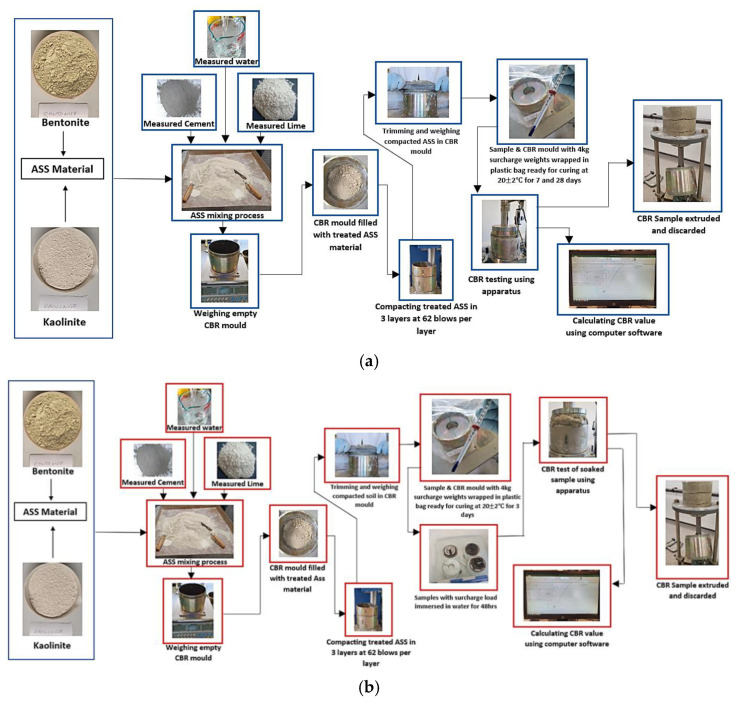
(**a**) The mixing and testing process of the treated-unsoaked CBR samples; (**b**) the mixing and testing process of the treated-soaked CBR samples.

**Figure 5:**
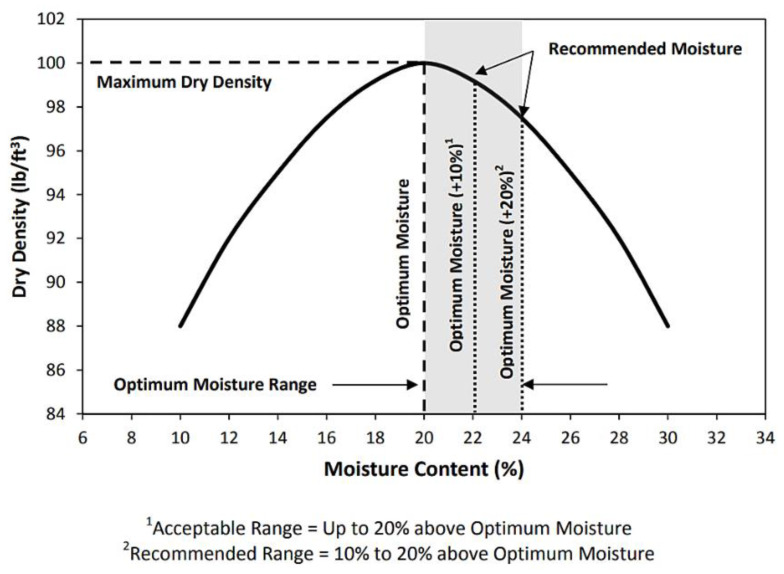
Moisture content control chart (Section 3/9 of [48]).

CBR test samples were prepared for all three ASS materials (treated and untreated). A total sample mass of 4 kg was required to achieve a fully compacted CBR mold. Based on total sample mass, bentonite and kaolinite at required proportions were weighed to form ASS 1 (25% bentonite, 75% kaolinite), ASS 2 (35% bentonite, 65% kaolinite) and ASS 3 (75% bentonite, 25% kaolinite). For treated ASS samples, various proportions of binders (8% lime and 20% cement) were selected based on a benchmark subgrade CBR value of 80%, capable of carrying a heavy traffic load of 5443 kg in accordance with the CBR method recommended by the California state of highways [46]. According to [44], a high-quality subgrade normally has a CBR value between 80–100% maximum. To achieve the target of 80% CBR, a range of cement and lime proportions were experimented with in subgrade mixtures using the intervals for cement and lime stabilization recommended by the Design Manual for Roads and Bridges (DMRB) HA 74/07 [47]. The cement and lime proportions were increased gradually (2%lime + 5%cement, 4%lime + 10%cement, 6%lime + 15%cement, etc.) until a CBR value of 80% was achieved for 8%lime 20%cement. The percentages of cement and lime were measured by weight of the total sample mass, and dry-mixed with the ASS materials until homogeneity was achieved. A measured amount of water was gradually added, based on the OMC for untreated ASS materials achieved during the proctor compaction testand mixed together to form a uniform mixture. Where the mixture looks and feels dry at OMC during the preparation of treated ASS materials due to the addition of binders (cement and lime), which imbibe water, a recommended amount of water within the range of 10–20% above the optimum moisture content (OMC) was added to the original moisture content (OMC) achieved during the proctor compaction test, in accordance with BS EN ISO 17892-1-2014 [35], BS EN 13286-47:2021 [43] and section 3/9 of the document [48]. A CBR mold (152 mm diameter × 178 mm high) was weighed on a scale without the collar, and the weight was recorded. The collar was later attached to the mold, and the uniformly mixed ASS material (4 kg) was divided into three equal parts and placed in layers in the mold during compaction. Each part was placed in the CBR mold and, with the help of a mechanical compactor, fitted with a 2.5 kg rammer; 62 blows were applied at different areas of the surface of each layer to ensure an even distribution of force. After the last layer was compacted, the mold containing the compacted sample was detached from the mechanical compactor. The collar was carefully removed, and the compacted ASS material was trimmed off using a pallet knife so that it was completely even with the top edge of the mold. The compacted ASS material with the mold and the base was weighed and the value recorded. At this stage, untreated ASS materials were tested for soaked and unsoaked CBR without curing. Treated (with binders) ASS samples and the mold were also wrapped in an airtight plastic bag, ready for curing at a room temperature of 20 ± 2 °C for 7 and 28 days, respectively. Figure 4a,b shows the mixing and testing process of the treated-unsoaked and treated-soaked CBR samples. Figure 5 shows the recommended OMC range recommended by [48].

CBR tests were carried out for all ASS material types (treated and untreated) to determine their bearing capacity in accordance with BS 13377-4:1990 [37] and BS EN 13286-47:2021 [43] using the Design Manual for Roads and Bridges (DMRB) CD 226 [49] and DMRB HA 74/07 [47] as a guide. The test was conducted to evaluate the subgrade strength of road and pavement by determining the ratio of force per unit area required to penetrate a soil mass with a standard circular plunger. 

CBR test samples were prepared and tested for all three types of untreated ASS material in accordance with relevant standards. The aim of conducting a CBR test on untreated expansive artificially-synthesized (ASS) materials was to determine its bearing capacity for use as subgrade materials without any modification, re-engineering, or treatment. Untreated ASS samples prepared for all three types of ASS materials were tested for CBR immediately after compaction without soaking. The same samples were prepared and soaked for 96 h (4 days) immediately after compaction at a temperature of 20 ± 2 °C at a level that the sample was fully immersed in water in accordance with BS EN 13286-47:2021 [43]. The idea of soaked CBR samples in this study was to investigate how the subgrade material would behave when the air voids in the sample are filled with water to simulate the effect and behavior of untreated expansive subgrade in the event of a flood. In this study, measured amounts of cement, lime and water were added to the ASS materials and mixed together until homogeneity. CBR test samples were prepared from the mix in accordance with the relevant standards. Samples were made for all three types of ASS material to be tested for unsoaked CBR after 7 and 28 days of curing at a room temperature of 20 ± 2 °C. Soaked CBR tests were conducted on samples soaked in water for 96 h (4 days) after 7 and 28 days curing at room temperature of 20 ± 2 °C in accordance with BS EN 13286-47:2021 [43]. The samples were fully immersed in water with a temperature of 20 ± 2 °C at a level that allows free access of the water to the top and bottom of the specimen. The idea of soaked CBR in this study was to investigate how treated subgrade material would behave when the air voids in the sample are filled with water to simulate the effect and behavior of stabilized or treated expansive road subgrade in the event of a flood.

### 2.2. Swell Test of Treated and Untreated ASS Materials

**Figure 6:**
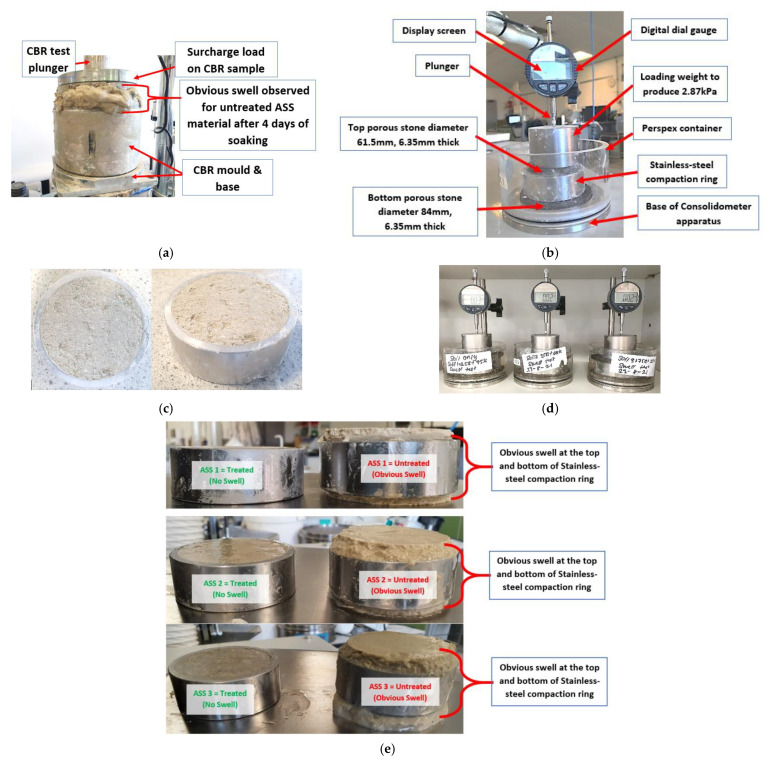
(**a**) Swell observed after soaking CBR untreated ASS samples; (**b**) consolidometer apparatus used in this study; (**c**) compacted swell samples in stainless-steel compaction ring; (**d**) swell set-up for treated and untreated ASS materials; (**e**) treated and untreated ASS samples after the swell test.

Even though a significant and obvious amount of swell was observed after the soaking process of untreated ASS samples, a separate swell test was conducted on both untreated and treated ASS materials. The swell behavior for untreated and treated expansive ASS materials was tested using the linear expansion measurement method in accordance with BS EN 13286-49:2004 [50]. At this stage, the amount of swell or expansion observed in the various ASS materials was measured using a self-contained basic swell consolidometer (BSC) apparatus. The apparatus included a stainless-steel compaction ring with a diameter of 2.42, two porous stones (top porous stone with diameter 61.5 mm, 6.35 mm thick, and bottom porous stone diameter 84 mm, 6.35 mm thick), a loading weight of 2.87 kPa and a dial gauge. Treated and untreated ASS material with a total mass of 100 g with or without a binder inclusive was weighed. A measured amount of water was added at OMC, mixed uniformly and used to prepare samples for the swell test by compacting the ASS mixture into the stainless-steel compaction ring. The compacted samples were placed in the consolidometer between the two porous stones, which were already soaked in water to allow water to seep through them immediately at the start of the test. A loading weight to produce 2.87 kPa was placed on top of the porous stone on the sample, and a dial gauge indicator was set to the initial sample height with the tip of the plunger touching the top of the loading weight. The dial gauge reading was set to zero, and the consolidometer was filled with water to begin the test. Untreated ASS materials were tested for swell immediately after the compaction without curing. Treated ASS subgrade materials were wrapped in cling film and cured at a room temperature of 20 ± 2 °C for 7 days before testing for swell. The aim of wrapping treated samples in cling film was to slow the rate of water evaporation and allow the binders (cement and lime) to chemically react in anticipation of reducing the swelling potential of the subgrade material. Dial gauge readings of the amount of swell were recorded daily for 28 days, and the data were analyzed to establish the swelling potentials of both treated and untreated ASS materials. Figure 6a–e shows the obvious swell after the soaked CBR test, including the consolidometer apparatus, compacted swell samples in the stainless-steel compaction ring and the swell set-up for treated and treated and untreated ASS samples after the swell test.

### 2.3. Microstructural Properties of Treated Subgrade Material

**Figure 7:**
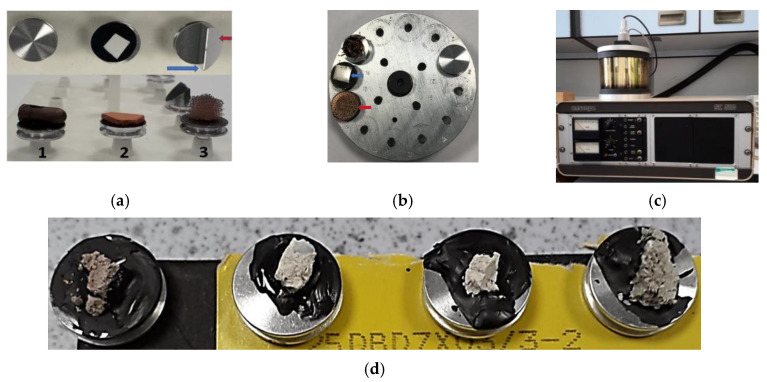
(**a**) (1) Samples are mounted on standard aluminum stubs 13 mm in diameter. The stub has a groove at the side to facilitate handling using forceps; (2) the aluminum stub with a double-sided adhesive black conductive carbon, tab. A, piece of filter paper has been cut to size and pressed down at the corners using forceps onto the tab; (3) this stub allows one to see a transverse view. The sample is mounted against the vertical face (blue arrow). The red arrow indicates a 45° angle face. (**b**) The stub holder for the SEM chamber. The blue arrow indicates a piece of metal that requires no further preparation. The red arrow shows a non-conductive sample that has been sputter coated with gold to make it conductive; (**c**) gold sputter coating unit; (**d**) treated artificially-synthesized subgrade (ASS) samples mounted and ready for the SEM and EDX analysis.

Microstructural properties are the properties that influence the physical properties of materials such as hardness, strength, high/low-temperature behavior, toughness, wear resistance and others [2]. Microstructural properties of materials can be determined in the laboratory by conducting a scanning electron microscopy (SEM) analysis, energy dispersive X-ray (EDX) analysis, radar detection, or a Mises strain test, among others [2]. A study conducted by [51] shows the SEM analysis results, which showed a high C-S-H gel development, resulting in high strength after adding 6% of limited leather waste ash (LLWA) in a mix. EDX patterns showed a high formation of calcium silicate hydrate (C-S-H) gel after 28 days when the expansive soil was stabilized or treated with 20% GGBS [52]. A combination of SEM and EDX analysis provides a better understanding of the surface material and the elemental composition of a sample, allowing for a more quantitative result offering the chemical composition and elemental investigation to provide a comprehensive evaluation of the results. In this study, scanning electron microscopy (SEM) and energy dispersive X-ray (EDX) analysis were conducted to determine the elemental composition of the stabilized or treated ASS materials, providing high-resolution imaging for identifying and evaluating the material’s surface structure, contaminants, flaws/corrosion and unknown particles and to determine the cause of failure and interaction between the materials. The SEM and EDX equipment used in this study include the FEI Quanta 650 field emission scanning electron microscope manufactured by Philips, supplied by Frost bank Tower, 401 Congress Avenue, Suite 1760, Austin, Texas USA and Oxford Instruments Aztec Energy EDX system using an X-Max 50 detector with a coverage area of 50 mm^2^ and a sputter Coater Emscope SC500 gold sputter coating unit manufactured by Oxford Instruments Inc. and supplied by Science House, Church Farm Business Park, Corston, Bath, UK. Figure 7a–d describes how samples are mounted, shows the stub holder for the SEM chamber and the Gold Sputter Coating Unit and shows the treated ASS samples ready for the SEM and EDX test.

## 3. Results and Discussion

### 3.1. Compaction and Atterberg Limits for Untreated ASS Materials 

**Figure 8:**
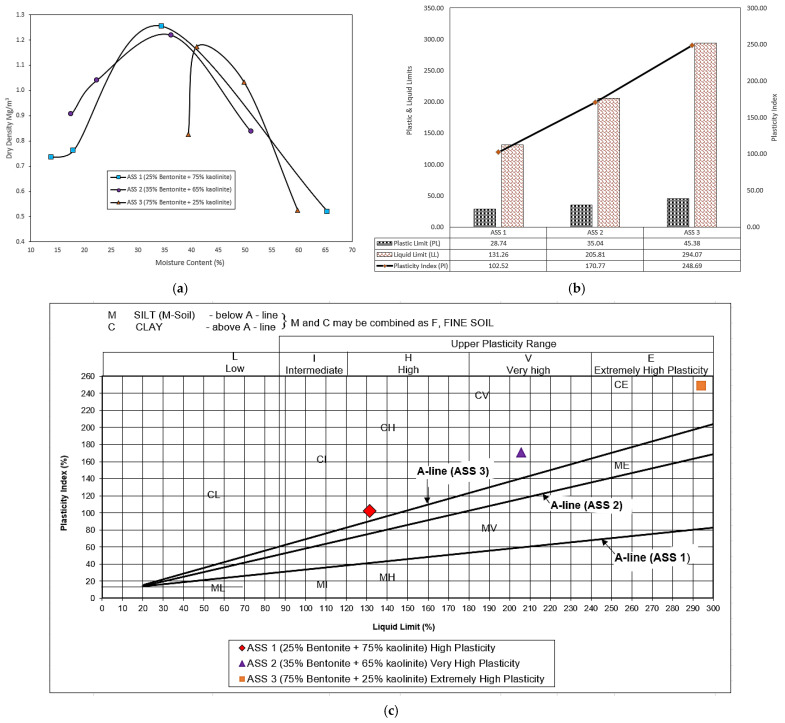
(**a**) Proctor compaction test results; (**b**) Atterberg limit test results against plasticity index; (**c**) plasticity index chat.

Results obtained after the proctor compaction and the Atterberg limit test show a high OMC, liquid limit (LL) and plastic limit (PL) recorded for ASS 3 (75% bentonite and 25% kaolinite), followed by ASS 2 (35% bentonite and 65% kaolinite) and ASS 1 (25% bentonite and 75% kaolinite). The increase and decrease in proctor compaction and Atterberg limit test results observed in the various ASS materials were a result of bentonite content in the mix. Bentonite clays are very expansive, with a high plasticity, and they imbibe a lot of water. After the preliminary test, the results showed a high plasticity for ASS 1 (25% bentonite and 75% kaolinite), a very high plasticity for ASS 2 (35% bentonite and 65% kaolinite) and an extremely high plasticity for ASS 3 (75% bentonite and 25% kaolinite), respectively. The gradual increase in plasticity as bentonite proportion increased is due to the high clay content in bentonite. According to [53], the gradual increase in the percentage of bentonite clay in a mix increases the plasticity index of the soil. Bentonite is highly water-absorbent and has high shrinkage and swell characteristics [54]. Figure 8 a–c shows the proctor compaction, Atterberg limit test results, Liquid and Plastic limit results against plasticity index and plasticity index chat for various ASS materials.

### 3.2. Moisture Content and Dry Density Test of CBR Sample

**Figure 9:**
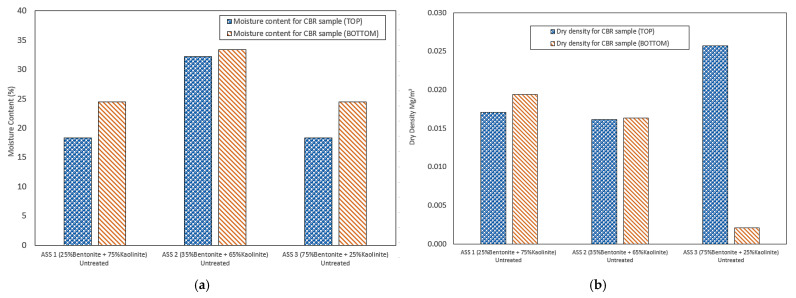
(**a**) Moisture content for the top and bottom of the CBR samples; (**b**) dry density for the top and bottom of the CBR samples.

The moisture content and dry density test were conducted on untreated CBR samples after testing by taking samples from the top and bottom of the CBR sample. This test was to determine the variation in moisture content and dry density at the top and bottom of the ASS sample immediately after compaction. The results showed a higher moisture content at the bottom of the CBR sample compared to the top of the sample. This could be a result of the settlement of water to the base of the sample due to the influence of gravity and the vertical force applied by the rammer. This is called gravitational water drain, which acts as a relative amount of water (capillary water) that is held between the soil particles due to the force of cohesion (surface tension that attracts water molecules to each other) and adhesion (the attraction of water molecules to other surfaces) that are stronger than gravity [55]. The highest moisture content was recorded at the top and bottom for ASS 2 (35% bentonite and 65% kaolinite), and ASS 1 and 3 recorded similar moisture content values for both the top and bottom of the sample. The highest dry density was recorded at the top of ASS 3, followed by a drastic reduction in dry density at the bottom of ASS 3 (75% bentonite and 25% kaolinite). ASS 2 recorded similar dry densities for both the top and bottom of the sample. Figure 9a,b shows the results of the moisture content and dry density for the top and bottom of the CBR samples.

### 3.3. California Bearing Ratio (CBR)

#### 3.3.1. Untreated ASS Materials

A CBR of 9% and 2% was recorded for the untreated and untreated-soaked ASS 3 (75% bentonite and 25% kaolinite) samples, representing the highest CBR values for untreated and untreated-soaked ASS materials. The results showed that the high plasticity subgrade (high amount of bentonite present in ASS 3) naturally exhibited a reasonably high bearing capacity, even though it may have high shrink-swell potentials. However, this naturally-high bearing capacity of bentonite can be affected by the addition of cement and or lime as binders in the mixture. ASS 1 (25% bentonite and 75% kaolinite) also achieved a CBR value of 8% for untreated-unsoaked and 0.9% for untreated-soaked ASS materials, followed by ASS 2 (35% bentonite and 65% kaolinite) with an untreated-unsoaked value of 5% and an untreated-soaked of 0.8%, respectively. Very low CBR values were observed for all soaked ASS samples, and this indicated that high-plasticity subgrade materials have a low bearing capacity when wet. However, a gradual increase in CBR values was observed for soaked samples with an increase in bentonite content. A naturally high CBR value was observed for mixtures with high bentonite content without treatment. However, it was observed that the addition of lime and cement reduced the natural bearing capacity of bentonite in the mixture. According to [56], pure bentonite has a high CBR value, which equates to 35.8%. This confirms the findings in this study that bentonite subgrade materials exhibit naturally high CBR values. Overall, the test results showed that the higher the presence of bentonite in ASS, the higher the CBR value and vice versa. Even though some value of CBR was recorded for ASS 1 soaked and ASS 2 soaked, these values were unacceptable for use in road construction, except for ASS 3 which hit the 2% mark. According to IAN73/06 [57], CBR values below 2% are not acceptable for use and will require some modification. Figure 9a shows the CBR results for the untreated ASS materials. 

#### 3.3.2. Treated ASS Materials

**Figure 10:**
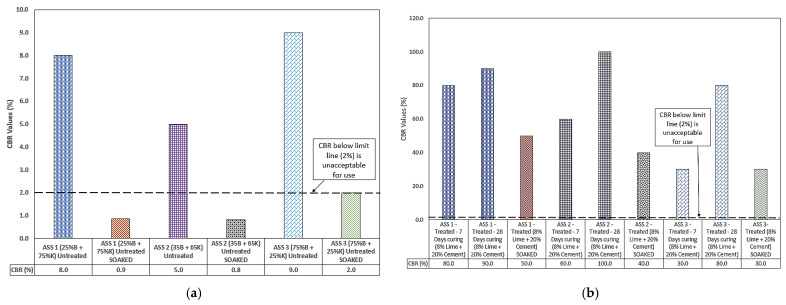
(**a**) Results for the untreated ASS materials, where B is bentonite and K is kaolinite; (**b**) treated artificially-synthesized subgrade.

The highest CBR value of 100% was recorded for ASS 2 (35% bentonite and 65% kaolinite), followed by a CBR of 90% for ASS 1 (25% bentonite and 75% kaolinite) and then 80% for ASS 3 (75% bentonite and 25% kaolinite) all after 28 days of curing. CBR values took a nosedive from 80% for ASS 1, to 60% for ASS 2 and 30% for ASS 3 all after seven days of curing. This showed a decrease in CBR value as bentonite content increased. Reasonably high CBR values were observed with an increase in curing age for ASS 2 and ASS 3, which were of a very high and extremely high plasticity index. ASS 2 at 28 days recorded the highest CBR values due to the presence of bentonite in the mix. As mentioned earlier in Section 3.3.1, the naturally-high bearing capacity of bentonite can be affected by the addition of lime and cement during the stabilization process. Thus, the reduction in the CBR value for ASS 3 could be due to the high presence of bentonite content in the mixture, and the high CBR value for ASS 1 and ASS 2 could be a result of low bentonite content in the mix, as they both recorded very high CBR values of 90 and 100%. According to [58], the unconfined compressive strength of lime-treated soil increased considerably because of a low content of bentonite added in the mixture. Reference [59] also stated that limited percentages of bentonite in a mix using lime as a binder is enough to improve the soil strength. This shows that high-plasticity bentonite subgrade materials exhibit a high bearing capacity when they are dry after they come in contact with water, and they are very weak when wet. This attribute of high-plasticity subgrade materials was responsible for the high CBR values and high swell observed in this study. According to [60], soils with a high plasticity index exhibit reasonable CBR values. Even though soils with a high plasticity index exhibit high strength when dry after coming into contact with water, their strength potential can be affected by the binders used during the stabilization process. Unlike untreated ASS samples, CBR values for soaked-treated samples decreased with an increase in bentonite (highly plastic clay) content because clays are weak in compression when wet. However, the CBR values achieved for the soaked-treated samples were good enough for use in road construction. A study conducted by [61] showed a reduction in CBR values from 15.41% to 3.56% as the bentonite content in a mix increased from 5%, 10%, 15%, 20% and 25% respectively. CBR values (8%) for untreated ASS 1 increased to 80% and 90% after treatment with cement and lime and after they were cured for 7 and 28 days. CBR values (5%) for untreated ASS 2 increased to 60% and 100% after treatment with cement and lime and after they were cured for 7 and 28 days. CBR values (9%) for untreated ASS 3 increased to 30% and 80% after treatment with cement and lime and after they were cured for 7 and 28 days. CBR values (0.9%) for untreated-soaked ASS 1 increased to 50% when soaked for four days after treatment with cement and lime and they were cured for seven days. CBR values (0.8%) for untreated-soaked ASS 2 increased to 40% when soaked for four days after treatment with cement and lime and they were cured for seven days. CBR values (2%) for untreated-soaked ASS 3 increased to 30% when soaked for four days after treatment with cement and lime and they were cured for seven days. This trend indicates a significant increase in CBR values with an increase in curing age after the subgrade materials were treated with cement and lime. Although an increase in CBR values was observed for untreated-soaked ASS samples after they were treated with cement and lime and they were cured and soaked for four days, a gradual reduction in CBR values in treated-soaked samples was observed for all ASS materials. This shows that the CBR values for subgrade materials with a high plasticity index can reduce when they are soaked in water for days. The CBR values achieved for treated-soaked and treated-unsoaked were above 2% and were suitable for use in road construction. This study has established that cement and lime have the ability to increase the bearing capacity of expansive road subgrade material. Overall, a decrease in CBR values was observed in treated ASS samples as bentonite content increased. Figure 10b shows the CBR results for the treated ASS materials.

### 3.4. Swell for ASS Materials 

#### 3.4.1. Untreated ASS Materials 

ASS materials began to swell after day 1, and ASS 1 and ASS 2 continued to swell until day 14, when no further swell was observed. ASS 3 continued to swell until day 3, with a slight reduction in swell on day 4 and a rise in swell at day 5, until no further swell was recorded. The highest swell percentage of 56.76% was recorded for ASS 3 (75% bentonite and 25% kaolinite), and the lowest swell percentage was 35.92% for ASS 1 (25% bentonite and 75% kaolinite), while ASS 2 (35% bentonite and 65% kaolinite) recoded a swell percentage of 40.52% all after 28 days of curing. This shows a high swell with an increase in bentonite content. This proves that extremely high and high plasticity subgrade materials exhibit very high swell potentials. According to standard practice, a subgrade swell > 2.5% is unacceptable and would require treatment or removal and replacement [62]. Hence, untreated ASS materials in this study did not meet the standard for use as subgrade material. Figure 10a shows the swell results for untreated ASS materials. The maximum swell values obtained at day 4 compared with four days soaked untreated CBR values shows that ASS 3, composed of very high bentonite (extremely high plasticity index) content, recorded a swell percentage value of 55%, with the highest CBR value of 2% for untreated-soaked ASS materials, followed by ASS 2 and ASS 1 of 33% swell, 0.8% CBR and 29% swell, 0.9% CBR, respectively. This shows that the higher the swell the lower the CBR value, and it confirms the statement made in this study about bentonite exhibiting some reasonable amount of CBR, even though they have very high swelling potentials.

#### 3.4.2. Treated ASS Materials

**Figure 11:**
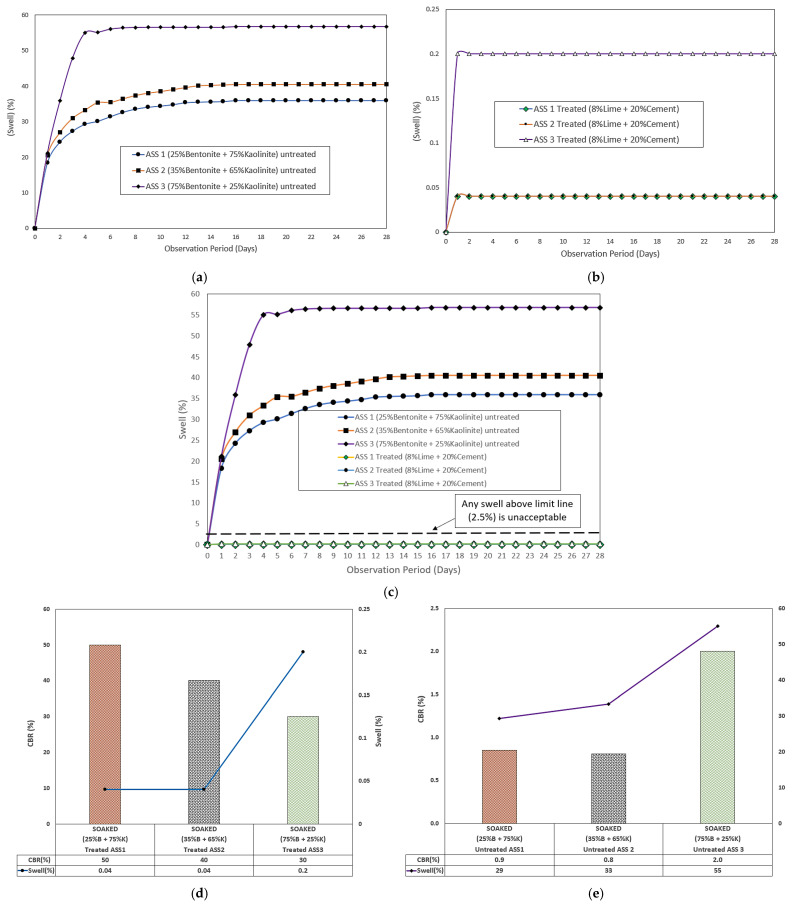
(**a**) Results for the untreated ASS materials; (**b**) results for the treated ASS; (**c**) combined swell result for untreated and treated ASS materials; (**d**) day 4 untreated-soaked CBR values against day 4 swell, where B = bentonite and K = kaolinite; (**e**) day 4 treated-soaked CBR values against day 4 swell, where B = bentonite and K = kaolinite.

The swelling potential of ASS reduced drastically from 55% for untreated ASS 3 (75% bentonite and 25% kaolinite) to 0.2% after treating ASS materials using cement and lime. The lowest swell value of 0.04% was recorded for ASS 1 and ASS 2, with high kaolinite contents, compared to ASS 3 with a high bentonite content. However, a swell value of 0.2% (even though acceptable) recorded for ASS 3 was the highest recorded for treated ASS samples due to the high amount of bentonite (extremely high plasticity index) content. This indicated very high swell potentials for subgrade materials with a high plasticity index. Swell values recorded for treated ASS materials in this study fell below the unacceptable 2.5% swell limit. Hence, all treated ASS materials in the study met the standard for use as subgrade materials in road construction. Figure 11b shows the results for treated ASS materials, and Figure 11c shows a combined swell result of both untreated and treated ASS materials for easy comparison. ASS 3, composed of a very high bentonite content, obtained the highest acceptable swell value of 0.2% against the lowest CBR value of 30% for treated-soaked ASS materials. This confirms the statement earlier made in this study, that binders (cement and lime) used during a road subgrade stabilization process can affect the bearing capacity of bentonite clay. After investigating the maximum swell values obtained after four days of soaking the treated CBR samples, a reduction in the CBR values with an increase in bentonite content, and an increase in swell values as bentonite content increased, was observed. Figure 11d and e shows the day 4 swell compared with the four-day soaked untreated CBR values and the day 4 swell compared with the four-day soaked treated CBR values.

### 3.5. Microstructural Properties of Treated Subgrade Material 

**Figure 12:**
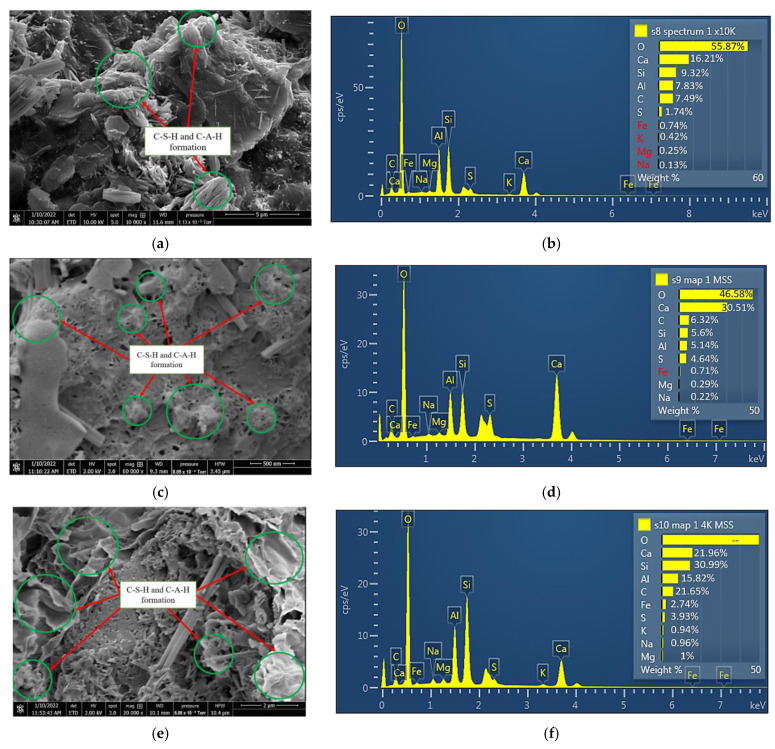
(**a**) SEM image results for ASS 1 after seven days of curing; (**b**) EDX results for ASS 1 after seven days of curing; (**c**) SEM image results for ASS 2 after seven days of curing; (**d**) EDX results for ASS 2 after seven days of curing; (**e**) SEM image results for ASS 3 after seven days of curing; (**f**) EDX results for ASS 3 after seven days of curing.

**Figure 13:**
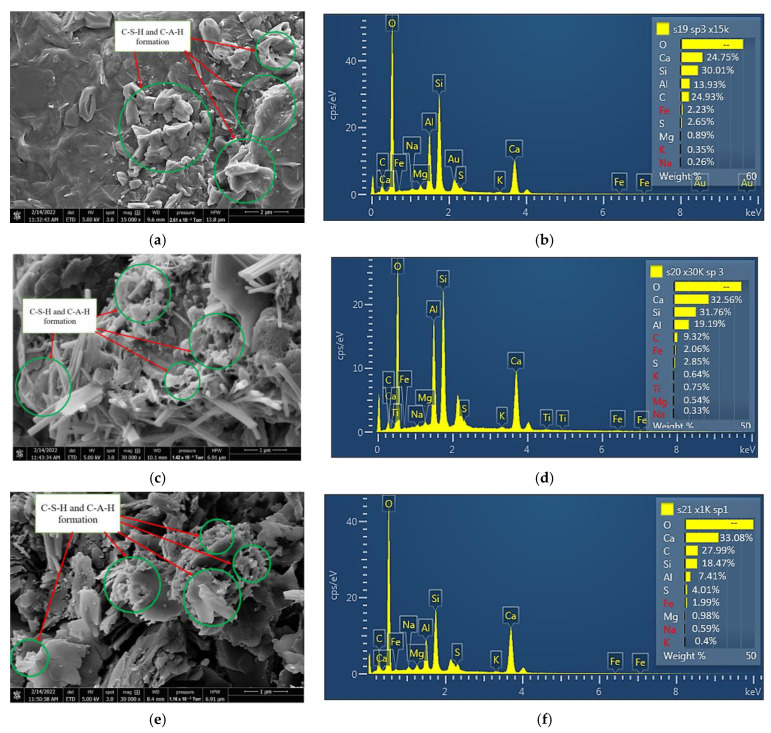
(**a**) SEM image results for ASS 1 after twenty-eight days of curing; (**b**) EDX results for ASS 1 after twenty-eight days of curing; (**c**) SEM image results for ASS 2 after twenty-eight days of curing; (**d**) EDX results for ASS 2 after twenty-eight days of curing; (**e**) SEM image results for ASS 3 after twenty-eight days of curing; (**f**) EDX results for ASS 3 after twenty-eight days of curing.

In this study, the SEM image and EDX results for the treated ASS 1 (25% bentonite + 75% kaolinite + 8% lime + 20% cement), ASS 2 (35% bentonite + 65% kaolinite + 8% lime + 20% cement) and ASS 3 (75% bentonite + 25% kaolinite + 8% lime + 20% cement) show the formation of calcium silicate hydrate (C-S-H) gel and calcium aluminate hydrate (C-A-H) gel with an increase in curing age. A clear presence of high Ca-Si-Al elements responsible for the formation of tobermorite gel was observed from the SEM map for the various chemical compositions in different areas of the ASS materials. Tobermorite is a chemical composed of calcium silicate hydrate mineral, with the chemical formula [Ca]_5 [Si]_6 O_16 [(OH)]_2.[4H]_2 O or [Ca]_5 [Si]_6 ([O,OH)]_18.[5H]_2 O, and it is responsible for the detoxification and strength gain in a mix. According to investigations conducted by [63], the relationship between the content of minerals formed in a mix and their detoxification efficiency shows that the formation of tobermorite helps to promote detoxification in a mix. This means the presence of toxic elements found in a mix due to the addition of binders (especially waste materials or industrial by-products, which can be very toxic due to leaching) can be detoxified due to the formation of a high amount of tobermorite (C-S-H and C-A-H gel) in the mix. During the hydration process in a cement/lime mix, cementitious products are released (C-S-H and C-A-H gel), which are responsible for the strength gain in the mixture [2,64]. The formation of C-S-H and C-A-H gel in this study acted as a binding agent responsible for the strength gain and the high CBR value of the subgrade materials. According to [2], Portland cement with lime in the presence of water forms hydraulic compounds: Portland cement + water → calcium silicate hydrate = Ca(OH)_2_ + CO_2_ → CaCO_3_ + H_2_O. Extra amounts of hydraulic cement are formed when the cement reacts with lime = Pozzolana + Ca(OH)_2_ + Water → C-S-H gel. At the end of seven days of curing, a formation of 16.21% calcium (Ca) was found in ASS 1, 30.51% calcium (Ca) in ASS 2 and 21.96% calcium (Ca) in ASS 3, respectively. All ASS samples cured for 28 days and exhibited a very high presence of C-S-H and C-A-H gel. At the end of 28 days of curing, the formation of 16.21% calcium (Ca) found in the seven-day ASS 1 increased to 24.75%, the 30.51% calcium (Ca) in the seven-day ASS 2 increased to 32.56% and the 21.96% calcium (Ca) for ASS 3 increased to 33.08%, respectively. This shows that the formation of C-S-H gel increased with an increase in curing age. The continuous formation of C-S-H gel with an increase in curing age within a pore structure can contribute to strength development in a mix; the higher the C-S-H gel content, the higher the strength in the samples [2,53]. Figure 12a–f and Figure 13a–f show the SEM image, mapping and EDX results for ASS 1, 2 and 3 at various points of the sample after 7 and 28 days of curing.

### 3.6. Road Pavement Thickness and Construction Depth Optimization

Road pavement thickness and construction depth optimization were conducted using the laboratory CBR values obtained for the various types of ASS materials in this study. The CBR values obtained in this study were analyzed with the aim of reducing the road pavement thickness and construction depth, while increasing the strength, durability and performance of the road pavement structure without compromising the relevant standards used in road design. According to [65], pavement thickness is determined by the subgrade strength, and it is good to make the subgrade as strong as possible. Road pavement thickness and construction depth optimization in this study was carried out in compliance with the CBR method recommended by the California state of highways for light traffic (3175 kg), medium traffic (4082 kg) and heavy traffic (5443 kg), respectively [46]. Relevant guidance, such as the Design Manual for Roads and Bridges (DMRB) CD 226 [49] and the Indian Roads Congress—IRC-37-2001 [45], used in flexible road pavement design, have shown that high CBR values are associated with a thinner road pavement thickness and a low CBR value results in a thicker pavement structure. The pavement thickness and construction depth determination chart recommended by the California state of highways were used to determine the pavement thickness and associated construction depth for various CBR values. 

**Figure 14:**
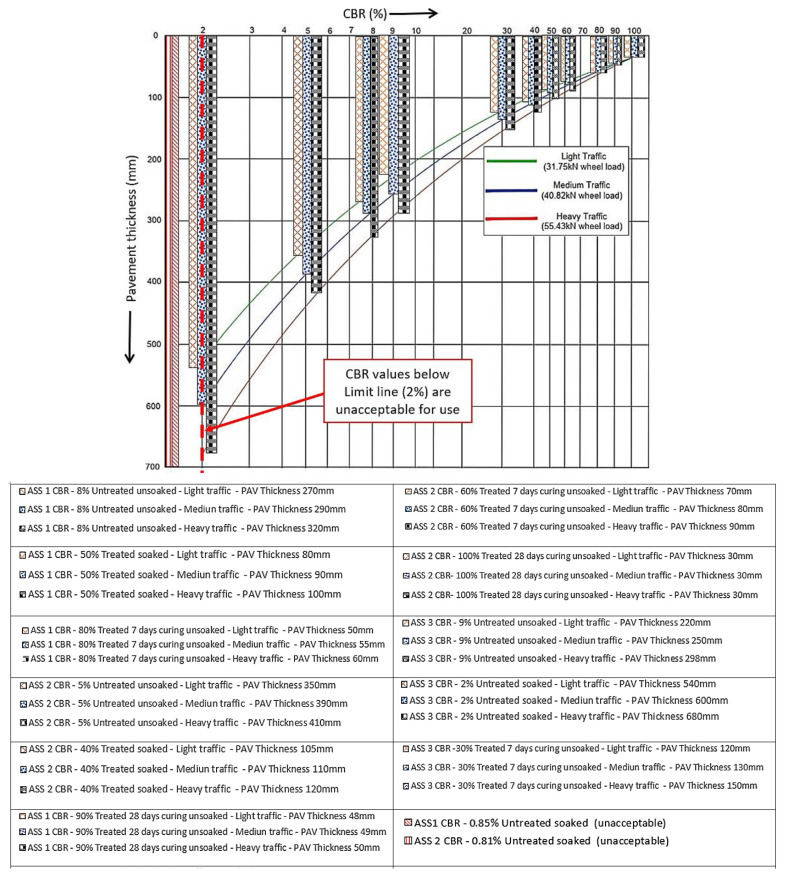
Road pavement thickness optimization using the chart for pavement thickness determination.

**Figure 15:**
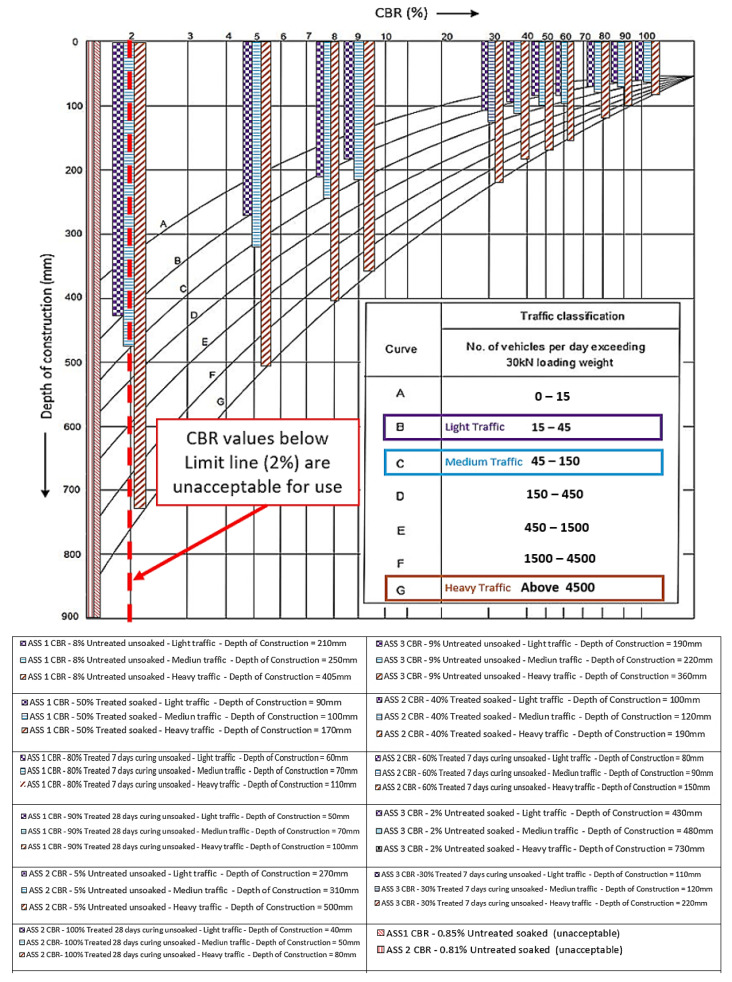
Pavement depths determination using the chart for construction depth determination.

**Figure 16:**
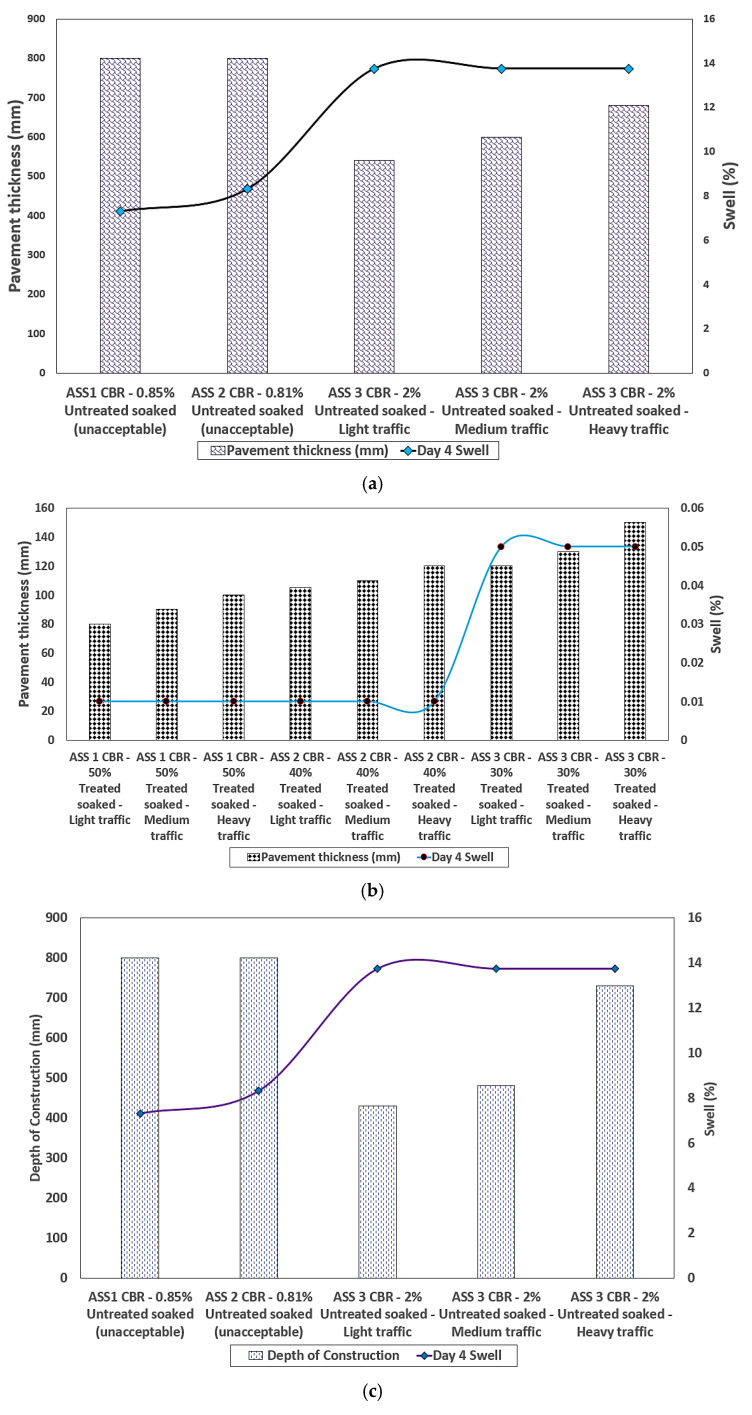

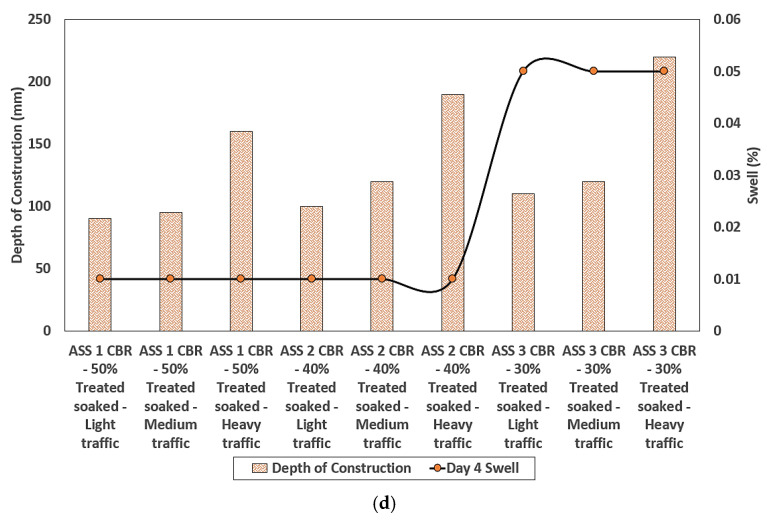
(**a**) Day 4 soaked pavement thickness against day 4 swell values for untreated ASS samples; (**b**) day 4 soaked pavement thickness against day 4 swell values for treated ASS samples; (**c**) day 4 soaked depth of construction against day 4 swell values for untreated ASS samples; (**d**) day 4 soaked depth of construction against day 4 swell values for treated ASS samples.

After pavement thickness optimization was conducted using the CBR values achieved in this study, it was observed that the pavement thickness reduced with an increase in CBR value. Hence, the higher the CBR value, the thinner the pavement thickness and vice versa. A significant difference in pavement thickness was observed between the lowest and the highest CBR value, and the pavement thickness for the CBR value deferred between the various traffic types. It was observed that a heavy traffic load required a thicker pavement, and a light traffic load required a thinner pavement, even though the same CBR value was used in their analysis. This is because heavy traffic requires thicker pavement to be able to carry a traffic load, reduce fatigue and control the deterioration of the road pavement. According to [66], road pavements are designed for predicted levels of traffic to control deterioration due to the accumulation of small amounts of damage caused by the passage of each vehicle. Pavement with less than about 180 mm of asphalt deforms at a high rate, but thicker pavement deforms at a lesser rate [66]. It is more economical to design road pavement for the existing subgrade capacity than to import or raise the subgrade support by using an extra-thick subbase [67]. The pavement thickness determination chart recommended by the California state of highways was used as a guide to determine light, medium and heavy traffic classifications for the construction depth determination. It was observed that the pavement depth of construction reduced as the CBR value increased, and the pavement depth of construction increased with an increase in traffic load. The highest pavement depth of construction was recorded for the CBR value of 2% for a heavy traffic load above 4500 kN, and the least pavement depth of construction was recorded for the CBR value of 100% for a light traffic load between 15–45 kN. A huge difference between the pavement depth of heavy traffic compared to light and medium traffic was observed. This is because heavy traffic required a robust road pavement to transfer the traffic load into the ground and reduce the rate of pavement deterioration. Very low CBR values of 2% and 0.81% were recorded for untreated-soaked ASS samples, resulting in thicker pavements. According to relevant guidance such as the IAN73/06 [57], CBR values less than 2% are unacceptable for use in road construction and would require modification or treatment to improve their engineering properties. Figure 14 and Figure 15 show the road pavement thickness and depths of construction optimization for CBR values achieved in this study. An increase in pavement thickness and construction depth was observed as the traffic load increased from light, medium to heavy traffic for both treated and untreated subgrade materials. It was also observed that ASS 3 (extremely high plasticity) recorded the thickest pavement with the lowest CBR value for treated and untreated ASS materials. An increase in swell and pavement thickness was observed as bentonite content increased in ASS materials, with ASS 3 (high bentonite content) recording the highest swell. Unacceptable CBR values for untreated ASS 1 and ASS 2 recorded a reasonably high swell; however, a high swell, pavement thickness and construction depth were recorded for the ASS 3 light, medium and heavy traffic loads. A gradual increase in pavement thickness and construction depth was observed with a rise in swell for ASS 1 to 3 light, medium and heavy traffic loads. Swell values increased drastically for treated-soaked ASS 3 for light, medium and heavy traffic loads (see Figure 14 and Figure 15). Figure 16 shows the day 4 soaked pavement thickness and the depth of construction against day 4 swell values for the treated and untreated ASS samples.

## 4. Conclusions

After conducting road pavement thickness and depth of construction optimization in this study, it was observed that the CBR values of the subgrade materials played a very vital role in determining the pavement thickness and construction depth of a road pavement structure. The conclusions arrived at after conducting the pavement thickness optimization for re-engineered artificially-synthesized expansive subgrade materials are as follows:

1. A reduction in pavement thickness with an increase in CBR value and a significant difference in pavement thickness between subgrade CBR values of 2% and 100% was observed. Pavement construction depth reduced as the CBR value increased, and pavement construction depth increased as traffic load increased. The deepest pavement depth value was recorded for the CBR value of 2% for heavy traffic, and the least pavement construction depth was recorded for the CBR value of 100% for the light traffic load.
2. Preliminary test results showed a high plasticity index, liquid limit and moisture content for the untreated subgrade materials with an increase in bentonite content in the mix. Swell values for all untreated CBR samples crossed the 2.5% unacceptable region, making them unsuitable for use as subgrade material, while all swell values for treated CBR samples fell below the 2.5% region, making them suitable for use in road construction. High swell values were recorded for samples with high bentonite content after 28 days of observation.
3. The engineering properties of the expansive subgrade materials were improved after treatment using lime and cement as additives. High swell values were recorded for samples composed of high bentonite content compared with samples with high kaolinite content. Swell potentials of ASS materials were reduced drastically from 56.76% to 0.04%, below the unacceptable subgrade swell value of >2.5%, after treatment using lime and cement as binders.
4. All untreated-soaked CBR samples fell below the 2% unacceptable region, making them unsuitable for use, while all treated-soaked and treated-unsoaked CBR samples crossed the acceptable 2% region, making them suitable for use in road construction. An increase in CBR values was observed as bentonite content increased for treated, untreated and soaked ASS samples. This shows that bentonite is strong in compression when it dries after coming in contact with water and is weak in compression when wet. It was established that the bearing capacity and strength of bentonite can be affected by binders (cement and lime) used during the stabilization process.
5. The study recommends that expansive subgrade materials found on-site during road construction should be stabilized or treated to improve their engineering properties inserted by removing and replacing them with imported materials. Stabilizing weak subgrade materials can reduce the overall road construction costs compared to the cost of removal and replacement of weak subgrades. Road pavement construction costs can also be reduced by achieving high CBR values after stabilization, resulting in thinner road pavement thickness.

## Figures and Tables

**Table 1:** The advantages of in situ treated subgrade and the disadvantages of the removal and replacement of subgrade.

Cement/Lime Treated Subgrade	
Less time, less cost and reduces environmental impact Improves the workability of the subgrade of the soil Reduces the plasticity and shrink/swell potential Reduces moisture susceptibility and migration Increases the speed of construction Increases the bearing capacity compared to untreated subgrade	Promotes soil drying Provides significant improvement to the working platform Uses onsite soil rather than removal and replacement Provides permanent soil modification (no leaching) Does not require mellowing period
**Subgrade Removal and Replacement**	
Time-consuming, Very costly and Greater environmental impact	

**Table 2:** Oxide and some of the chemical composition of the bentonite and kaolinite clays.

Oxide	SiO_2_	Al_2_O_3_	Fe_2_O_3_	FeO	MgO	CaO	K_2_O	SO_3_	TiO_2_	Na_2_O	BaO	Cr_2_O_3_	Trace	L.O.I
Bentonite clay	63.02	21.08	3.25	0.35	2.67	0.65	-	-	-	2.57	-	-	0.72	5.64
Kaolinite clay	48.5	36.0	1.00	-	0.30	0.2	2.15	-	0.06	0.15	-	-	-	11.7
Cement (%)	20	6.0	3.0	-	4.21	63	-	2.30	-	-	-	-	-	0.80
Lime (%)	3.25	0.19	0.16	-	0.45	89.2	0.04	2.05	-	-	-	-	-	-

**Table 3:** Mineralogical composition of bentonite and kaolinite.

Mineralogy	Kaolinite (%)	Quartz (%)	Na-Montmorillonite (%)	Feldspar (%)	Calcite (%)	Micaceous Materials (%)	Organic Material (%)
Chemical formula	Al_2_Si_2_O_5_OH)_4_	SiO_2_	Na_33_Mg_33_Al_1.67_Si4O_10_(OH)_2_	CaAlSi_3_O_8_	CaCO_3_	-	-
Bentonite clay	0	18	20	0	3	0	0
Kaolinite clay	84	48	0	1	0	13	2

**Table 4:** Consistency limits and physical properties of kaolinite and Bentonite.

Properties	Kaolinite Clay	Bentonite Clay
Consistency limits		
Liquid limit w_L_ (%)	59	310
Plastic limit w_P_ (%)	28	49
Plasticity index I_P_ (%)	31	261
Other physical properties		
Water absorption	-	16.0
Density ℃	2.4	2.5 at 20 ℃
Bulk density glcc	-	1.18
Maximum dry density (kN/m^3^)	14.21	11.26
Relative density g/cm^3^	1.8	2.7
Solubility in water (g/L)	Insoluble	Insoluble
Natural moisture content (%)	28	14

## Data Availability

Data can be obtained from corresponding authors upon reasonable request.
